# Prognostic value of nutritional status in patients with human immunodeficiency virus infection-related lymphoma

**DOI:** 10.3389/fnut.2022.1050139

**Published:** 2022-11-10

**Authors:** TingTing Liu, RenZhi Hu, Jing Lv, Qin Luo, LuXiang Xu, ChaoYu Wang, Jun Liu, ZaiLin Yang, LingLi Xu, Yao Liu

**Affiliations:** ^1^Chongqing Key Laboratory of Translational Research for Cancer Metastasis and Individualized Treatment, Department of Hematology-Oncology, Chongqing University Cancer Hospital, Chongqing, China; ^2^Chongqing Key Laboratory of Translational Research for Cancer Metastasis and Individualized Treatment, Department of Personnel Section, Chongqing University Cancer Hospital, Chongqing, China

**Keywords:** nutritional status, lymphoma, human immunodeficiency virus, prognosis, CONUT score

## Abstract

**Objective:**

To investigate the predictive value of nutritional status on the prognosis of patients with human immunodeficiency virus (HIV) infection-related lymphoma.

**Materials and methods:**

A total of 149 patients with HIV infection-related lymphoma who were admitted to our hospital from August 2012 to May 2022 were selected as research subjects. Based on the patient prognosis, they were divided into a poor prognosis group (*n* = 30) and a good prognosis group (*n* = 119). General data from patients in both groups were collected, and the nutritional status of the patients was evaluated using the Controlling Nutritional Status (CONUT) score. Factors affecting the prognosis of HIV infection-related lymphoma were analyzed using univariate and multivariate analyses, and a prediction model was developed based on the analyzed factors. The receiver operating characteristic (ROC) curve was used to analyze the prediction model of the CONUT score alone and included the CONUT score in the prognosis of patients with HIV infection-related lymphoma. The predictive value of the data was assessed, and a survival curve was drawn to compare the survival of patients with different nutritional statuses.

**Results:**

There were significant differences in age, B symptoms, treatment conditions, International Prognostic Index (IPI), pathological stage, Eastern Collaborative Tumor Group physical status score (ECOG PS), CD4+ cell count, β2 microglobulin, and lactate dehydrogenase (LDH) between the poor prognosis group and the good prognosis group (*p* < 0.05). The CONUT score of the poor prognosis group was higher than that of the good prognosis group, and the difference was statistically significant (*p* < 0.05). A univariate analysis demonstrated that the age, B symptoms, treatment status, IPI, pathological stage, ECOG PS, CD4+ cell count, β2 microglobulin, LDH, and CONUT score were prognostic factors for patients with HIV infection-related lymphoma (*p* < 0.05). The results of a multivariate regression analysis demonstrated that the age, B symptoms, treatment status, IPI, pathological stage, ECOG PS, and CONUT score were independent risk factors for the prognosis of patients with HIV infection-related lymphoma (*p* < 0.05). The prediction model was constructed according to the multivariate Cox regression analysis results. The model formula was as follows: Logit(*p*) = −10.687 + 1.728 × age + 1.713 × B symptoms + 1.682 × treatment status + 1.810 × IPI + 1.643 × pathological stage + 1.584 × ECOG PS + 1.779 × CONUT score. The ROC curve was used to analyze the predictive value of the CONUT score alone and the predictive model including the CONUT score on the prognosis of patients with HIV infection-related lymphoma. The predictive value of the prognosis of patients with tumors was higher (*p* < 0.05). According to the results of the ROC curve analysis, the patients were divided into a high CONUT group (CONUT > 6.00 points, *n* = 31) and a low CONUT group (CONUT ≤ 6.00 points, *n* = 118) based on the Optimum threshold of the CONUT score. The survival curve showed that the survival rate of the high CONUT group was lower than that of the low CONUT group (*p* < 0.05).

**Conclusion:**

The poor prognosis of HIV infection-related lymphoma is related to nutritional status, which is an independent risk factor affecting the prognosis of patients and can be used as a practical indicator to predict the prognosis of patients.

## Introduction

Human immunodeficiency virus (HIV) infection-associated lymphoma is a heterogeneous malignancy that encompasses multiple types of lymphoma, such as non-Hodgkin’s lymphoma (NHL) and Hodgkin’s lymphoma (HL). Studies have shown that, compared with the general population, the probability of lymphoma in patients with HIV-infection is significantly increased. Before the popularization of antiretroviral therapies, the relative risk of NHL in patients with HIV-infection was 100–500 times higher than in the general population, and the relative risk of developing HL was significantly higher than in the general population. The relative risk of HL is 5–26 times that of the general population in patients with HIV (1). Diffuse large B cell lymphoma (DLBCL) and primary central nervous system lymphoma (PCNSL) are the most common HIV infection-associated lymphomas, the incidence of HIV infection was 453 and 233 per 100,000 patients, respectively (2), and its main clinical manifestations were fever of unknown cause, lymphadenopathy, and abdominal pain (3), which seriously affected the health and quality of life of patients. The occurrence of HIV-associated lymphoma is related to immunosuppression, inflammatory cascade, and cytokine imbalance caused by HIV invasion (4). At present, continuous clinical research is being performed on HIV infection-associated lymphoma. It has great heterogeneity, and different patients have different prognoses. Studies on HIV-negative lymphoma have found that nutritional status indicators, such as body mass index (BMI), serum albumin (ALB), serum prealbumin (PA), and hemoglobin (Hb), can be used as prognostic risk factors for lymphoma (5). There are no such studies for HIV-associated lymphomas. In this study, to determine whether nutritional status is associated with the prognosis of patients with HIV-infected lymphoma, the predictive value of the Controlling Nutritional Status (CONUT) score was analyzed using a screening tool that comprehensively assessed the nutritional status of patients. This study analyzes the prognosis and survival of patients with different scores and provides a reference for selecting prognostic assessment tools for these patients in clinical practice.

## Research subject

### Clinical date

In total, 149 patients with HIV-infection-associated lymphoma admitted to our hospital between August 2012 and May 2022 were selected for the study. This included 126 men and 23 women, aged 25–80 years, with a mean age of 53.12 ± 15.11 years. Pathological types included: DLBCL in 79 cases, Burkitt lymphoma (BL) in 30 cases, HL in 13 cases, Plasmablastic lymphoma (PBL) in 8 cases, T-cell lymphoma (TCL) in 10 cases, and others in 9 cases. The patients and their families were informed of the study and signed an informed consent form.

### Inclusion and exclusion criteria

Inclusion criteria were as follows: (1) all patients showed positive serum anti-HIV antibody tests, a clear diagnosis of HIV infection, and pathological histological confirmation of HIV infection-associated lymphoma; (2) patients with complete clinical data; (3) patients with expected survival of more than 6 months; (4) patients aged greater than 18 years; and (5) voluntary participation, with informed patients and family members.

Those patients were excluded as follows: (1) non-HIV infection-associated lymphoma; (2) combination of other types of malignancies; (3) combination of acute and chronic infectious diseases; (4) inability to communicate; and (5) death due to reasons other than the disease during follow-up.

### Methods

#### Prognostic evaluation

All patients were followed up by outpatient or telephone until July 2022, with a median follow-up time of 44 months. According to different prognoses, patients were divided into a poor prognosis group (*n* = 30) and a good prognosis group (*n* = 119). The poor prognosis group was defined as patients with PD and death from any cause. The good prognosis group was defined as the patients who achieved stable disease (SD), partial response (PR), and complete response (CR). Computed tomography (CT) or 18F-fluorodeoxyglucose positron emission tomography/computed tomography (PET/CT) was performed for radiological evaluation. The 2007 revised Cheson criteria were used to define CR, PR, SD, and progressive disease (PD).

#### Clinical data collection

General data of patients in the poor and good prognosis groups were collected. This included age (≥60 years or <60 years), gender (male or female), time from HIV infection to lymphoma diagnosis (meanwhile/HIV infection 1–3 years/HIV infection >3 years), disease type (DLBCL/BL/HL/PBL/TCL/others), tumor diameter (>5 or ≤5 cm), B symptoms (with/without), treatment condition (withdrawal of treatment/receiving 1–2 cycles of chemotherapy/receiving over two cycles of chemotherapy), chemotherapy regimen (R-epoch/EPOCH), Lymphoma International Prognostic Index (IPI, 0–2/3–5), pathological stage (stage i–ii / stage iii–iv), extranodal involvement (with/without), Eastern Collaborative Tumor Group physical status score (ECOG PS, >2 points/ ≤ 2 points), CD4+ cell count (≥200 × 106/L/ < 200 × 106/L), β2 microglobulin (elevated/normal), and lactate dehydrogenase (LDH) (elevated/normal).

#### Nutritional status assessment

The CONUT score is used to evaluate the nutritional status of patients. The CONUT was first proposed by Ignacio de Ulibarri et al. (6), and includes serum albumin (Alb), total cholesterol (TC), and total blood Lymphocytes count (LYM), which involves protein reserves, calorie consumption, and immune defense. It can be applied to all populations. The specific evaluation criteria are shown in Table 1.

**TABLE 1:** The controlling nutritional status (CONUT) scoring criteria.

Projects	Score rank			
Alb (g/dl)	3.5∼4.5 (0)	3.0∼3.49 (2)	2.5∼2.90 (4)	<2.5 (6)
TC (mg/dl)	>180 (0)	140∼180 (1)	100∼139 (2)	<100 (3)
LYM (number/ml)	>1,600 (0)	1,200∼1,599 (1)	800∼1,199 (2)	<800 (3)
CONUT score	normal (0∼1)	Mild (2∼4)	Medium (5∼8)	Severe (9∼12)

### Statistical analysis

The Statistical Product and Service Solutions (SPSS) 22.0 software was used for data analysis and the Kolmogorov–Smirnov test was applied to test whether the data conformed to a normal distribution. The measurement data conforming to a normal distribution were described using the mean ± standard deviation ($\bar{x}\pm s$), and a comparison between the two groups was made using the independent samples *t*-test, which did not conform to a normal distribution and was expressed using M(Qn) after natural logarithm transformation using the non-parametric test. Count data expressed as a rate (%) was used as a χ^2^-test. Receiver operating characteristic (ROC) curves were used to analyze the predictive value of the CONUT score on the prognosis of patients with HIV infection-associated lymphoma. Correlations were analyzed using the Pearson correlation analysis and factors affecting the prognosis of patients with HIV infection-associated lymphoma were analyzed using logistic regression. The log-rank test was used for the survival analysis. The value of *p* < 0.05 was considered a statistically significant difference.

## Results

### Comparison of clinical information between the poor prognosis group and the good prognosis group

The differences between the poor prognosis group and the good prognosis group were statistically significant (*p* < 0.05) in age, symptoms B, treatment status, IPI, pathological stage, ECOG PS, CD4+ cell count, β2 microglobulin, and LDH. The CONUT score of nutritional status in the poor prognosis group was higher than that in the good prognosis group, and the difference was statistically significant (*p* < 0.05) (Table 2).

**TABLE 2:** Comparison of clinical data between the poor prognosis group and the good prognosis group.

General information		Poor prognosis group (*n* = 30)	Good prognosis group (*n* = 119)	χ^2^/*t*	*P*
Age (year)		65.53 ± 6.78	54.74 ± 4.92	9.893	<0.001
Gender	Male	24 (80.00)	102 (85.71)	0.599	0.439
	Female	6 (20.00)	17 (14.29)		
Time from HIV infection to diagnosis of lymphoma	Meanwhile	21 (70.00)	56 (47.06)	5.549	0.062
	1∼3 year	8 (26.67)	48 (40.34)		
	>3 year	1 (3.33)	15 (12.61)		
Type of disease	DLBCL	16 (53.33)	63 (52.94)	0.031	0.860
	None-DLBCL	15 (50.00)	55 (46.22)		
Tumor diameter	>5 centimeter	10 (33.33)	46 (38.66)	0.289	0.591
	≤5 centimeter	20 (66.67)	73 (61.34)		
Symptoms B	With	19 (63.33)	41 (34.45)	9.123	0.003
	Without	11 (36.67)	78 (65.55)		
Status of treatment	≥4 cycles of chemotherapy	23 (76.67)	52 (43.70)	14.708	<0.001
	<4 cycles of chemotherapy	7 (23.33)	67 (56.30)		
Chemotherapy regimens	R-EPOCH	19 (63.33)	78 (65.55)	0.076	0.782
	EPOCH	11 (36.67)	41 (34.45)		
IPI	0∼2	7 (23.33)	83 (69.75)	31.794	<0.001
	3∼5	23 (76.67)	36 (30.25)		
Pathological staging	I∼II	5 (16.67)	90 (75.63)	53.258	<0.001
	III∼IV	25 (83.33)	29 (24.37)		
Extramodular involvement	With	20 (66.67)	68 (57.14)	1.158	0.281
	Without	10 (33.33)	51 (42.86)		
ECOG PS	>2	18 (60.00)	43 (36.13)	7.270	0.007
	≤2	12 (40.00)	76 (63.87)		
CD4+ cell count (× 10^6^/L)	≥200	27 (90.00)	64 (53.78)	19.496	<0.001
	<200	3 (10.00)	55 (46.22)		
β2 microglobulin	Elevated	25 (83.33)	72 (60.50)	8.116	0.004
	Normal	5 (16.67)	47 (39.50)		
LDH	Elevated	24 (80.00)	59 (49.58)	13.063	<0.001
	Normal	6 (20.00)	60 (50.42)		
COUNT score (point)		7.67 ± 1.55	3.13 ± 0.55	26.250	<0.001

### Regression analysis of factors influencing the prognosis of patients with human immunodeficiency virus infection-associated lymphoma

The prognosis of patients with HIV infection-associated lymphoma was used as the dependent variable (good = 0, poor = 1) and clinical data and nutritional status were used as independent variables for the regression analysis. Univariate analysis showed that the age, symptoms B, treatment status, IPI, pathological stage, ECOG PS, CD4+ cell count, β2 microglobulin, LDH, and CONUT score were factors influencing the prognosis of patients with HIV infection-associated lymphoma (*p* < 0.05). Multi-factor regression analysis showed that age, B symptoms, treatment status, IPI, pathological stage, ECOG PS, and CONUT score were independent risk factors affecting the prognosis of patients with HIV infection-associated lymphoma (*p* < 0.05) (Table 3).

**TABLE 3:** Regression analysis of factors influencing the prognosis of patients with human immunodeficiency virus (HIV) infection-associated lymphoma.

	Single factor analysis			Multi-factor analysis		
	*HR*	95% CI	*P*	*HR*	95% CI	*P*
Age	3.889	1.378∼7.892	0.019	1.897	1.007∼7.421	0.023
Time from HIV infection to diagnosis of lymphoma	1.142	0.719∼2.341	0.478	–	–	–
Type of disease	1.228	0.880∼2.338	0.335	–	–	–
Tumor diameter	1.392	0.823∼1.984	0.398	–	–	–
Symptoms B	1.421	0.653∼1.778	0.000	2.176	1.029∼10.092	0.012
Status of treatment	1.320	0.558∼2.012	0.003	5.082	2.341∼16.980	0.000
Chemotherapy regimens	1.332	0.576∼1.980	0.228	–	–	–
IPI	3.421	2.341∼7.998	0.003	3.018	1.112∼15.672	0.008
Pathological staging	2.391	1.372∼8.562	0.019	3.229	2.089∼13.415	0.000
Extramodular involvement	0.617	0.332∼0.786	0.384	–	–	–
ECOG PS	4.138	1.036∼7.128	0.017	2.798	1.002∼10.872	0.010
CD4+ cell count	5.668	2.342∼10.896	0.002	–	–	–
β2 microglobulin	4.634	2.337∼18.971	0.014	–	–	–
LDH	4.576	1.243∼16.998	0.004	–	–	–
COUNT score	5.394	1.045∼13.445	0.016	4.386	3.421∼11.097	0.000

“–” Indicates no date available.

### Predictive value of indicators for the prognosis of patients with human immunodeficiency virus infection-associated lymphoma

**FIGURE 1:**
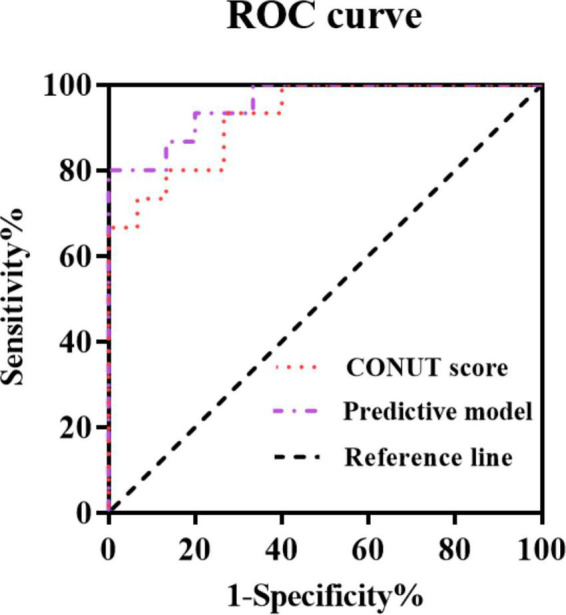
Curves for predicting the prognosis of patients with human immunodeficiency virus (HIV) infection-associated lymphoma by indicators.

A predictive model was constructed based on the results of the multi-factor Cox regression analysis in Table 4, according to the following model formula: Logit (*p*) = −10.687 + 1.728 × age + 1.713 × B symptoms + 1.682 × treatment status + 1.810 × IPI + 1.643 × pathological stage + 1.584 × ECOG PS + 1.779 × CONUT score. The ROC curve was used to analyze the predictive value of the CONUT score alone and the predictive model including the CONUT score for the prognosis of patients with HIV infection-associated lymphoma (Table 4 and Figure 1).

**TABLE 4:** Efficacy of indicators to predict the prognosis of patients with human immunodeficiency virus (HIV) infection-associated lymphoma.

	AUC	Sensitivity (%)	Specificity (%)	95% CI	*P*	Optimum threshold
COUNT score	0.910	85.61	75.55	0.786∼0.943	0.000	6.00
Predictive models	0.956	90.87	77.86	0.799∼0.985	0.000	–

### Correlation analysis between controlling nutritional status score and patients’ prognosis survival

**FIGURE 2:**
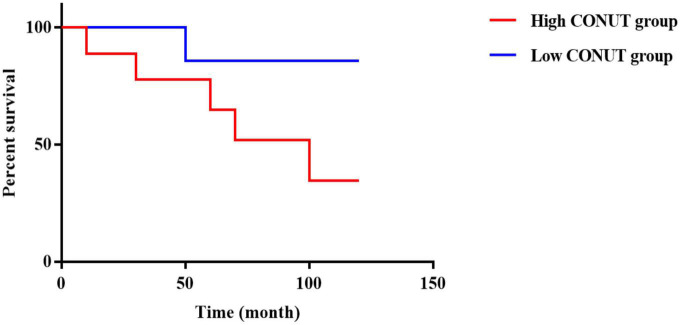
The high Controlling Nutritional Status (CONUT) group and the low CONUT group survival curve.

According to the results of the ROC curve analysis, patients were divided into a high CONUT group (CONUT > 6.00 score, *n* = 31) and a low CONUT group (CONUT ≤ 6.00 score, *n* = 118) using the optimum threshold of CONUT score as the basis for grouping. The plotted survival curve showed that the survival rate of the high CONUT group was lower than that of the low CONUT group (*p* < 0.05) (Figure 2).

## Discussion

Studies have shown (7, 8) that after HIV infection, a decrease in the number of CD4+ cells in the host is accompanied by an increase in the number of CD8+ cells and abnormal activation. This increases the production of tumor necrosis factor α, interferon, and other related cytokines. Additionally, the abnormal proliferation and activation of B cell after HIV infection and the abnormal increase in the body’s non-specific gamma globulin compromise the body’s immune system. The continued attack on CD4+ T lymphocytes by HIV leads to a continuous decrease in the number of CD4+ T lymphocytes, which increases the risk of malignancies, most commonly HIV-associated lymphoma (9).

In recent years, it has been reported (10, 11) that approximately 30% of patients with malignancies exhibit malnutrition, and it is believed that patient malnutrition is influenced by factors, such as altered body metabolism, immune cascade, and tumor-related anorexia, while patients who develop malnutrition have weakened immune defenses, such as macrophage function, natural barriers, and humoral immunity, increasing the occurrence of treatment insensitive events. The prognosis of patients with HIV-associated lymphoma is related to several factors, of which malnutrition is an independent risk factor. Therefore, early assessments of the patient’s nutritional status are important for improving the patient’s prognosis.

The CONUT score is currently the most commonly used sensitive index to evaluate the nutritional status of patients in clinical practice. It can comprehensively reflect the nutritional status of patients by assessing their Alb, TC, LYM, and other aspects, such as protein reserves, calorie consumption, and immune defense status (12, 13). The CONUT score can be used to comprehensively assess the nutritional status of patients with non-HIV-infected lymphoma and can be a sensitive predictor of prognosis in patients with lymphoma (14, 15). As the most commonly used clinical index in the CONUT score to evaluate the nutritional status of the patient’s body functions, Alb is reduced in patients with lymphoma. The possible mechanisms for Alb reduction could be (16–18), (1) increased catabolism of Alb, (2) loss of Alb due to changes in vascular permeability from the rapid increase of excessive inflammatory factors in the peripheral blood of patients as a result of local inflammatory cascade after the development of lymphoma. TC, which is an important component of the cell membrane, is associated with the proliferation, migration, and immune response of malignant tumor cells and can promote the recognition of tumor cells by immune cells by enhancing the antigen presentation function of monocytes (19–21). As a member of the inflammatory cell family, LYM is involved in altering the tumor microenvironment. Patients with low LYM levels were reported to have a worse prognosis than those with high LYM levels (22–24). In this study, we further explored the factors that affect the development of HIV infection-associated lymphoma. Both the univariate and multivariate analyses suggest that the CONUT score is an independent risk factor for patient prognosis, probably because the CONUT score can be a comprehensive assessment of changes in Alb, TC, and LYM levels in patients, and the poor nutritional status can have a negative impact on patient prognosis through inflammation or alterations in immune systems. Based on the above studies, we used the CONUT score for the first time in the assessment of the nutritional status of patients with HIV infection-associated lymphoma. Results showed that patients with poor prognoses had significantly higher CONUT scores than those with good prognoses, which may be explained by reduced Alb and LYM levels after lymphoma development. Impaired immune response in the tumor microenvironment, abnormal TC, reduced CONUT scores, weakened immune defense system, immune imbalance, and inflammatory cascade phenomena ultimately lead to malnutrition in patients. Our results are consistent with previous studies (25).

Current developments in nutritional pharmacology have raised the role of nutritional support to a new level in systematic therapies. The 2009-Guidelines for the treatment of clinical oncology patients with nutritional support published by the American Society for Parenteral Enteral Nutrition suggested that nutritional support does not need to be routinely used during surgery, chemotherapy, and radiation therapy for oncology patients. However, if malnutrition or risk of malnutrition exists, nutritional support is necessary and correct, and failure to correct the nutritional status of the body will increase the risk of poor prognosis (26). Our results in this study showed that in addition to age, B symptoms, treatment status, IPI, pathological stage, and ECOG PS, the nutritional status of the body was also an independent risk factor for poor prognosis, and the prognostic survival rate of patients with poor nutritional status was significantly lower. This suggests that nutritional status can decrease the survival rate of patients. The reason could be that for patients with HIV infection-associated lymphoma, the body needs more nutrition to repair the damage caused by tumor lesions, so it is easily accompanied by malnutrition, which in turn reduces the body’s resistance to disease and seriously affects patients’ recovery and even survival. Therefore, it is recommended that in clinical practice, attention could be paid to the nutritional status of patients, and parenteral (for patients with concomitant gastrointestinal dysfunction) or enteral (for patients with normal gastrointestinal function) nutritional supplementation should be given to patients to reduce the occurrence of prognostic adverse events.

This study used the CONUT score to assess the nutritional status of the patients, which is different from other nutritional status scoring systems since this score also evaluates TC indicators along with Alb and LYM. This can indirectly affect the immune response of the tumor microenvironment of the patients and can better reflect the prognosis of the patients. However, this study also has certain shortcomings, such as using the CONUT score only as an indicator of nutritional status, and not analyzing the impact of other nutrition-related biochemical indicators on the prognosis of patients. Therefore, follow-up studies incorporating multiple nutrition-related indicators as well as a long-term follow-up are needed to fully analyze the impact of nutritional status on prognosis.

In summary, a poor prognosis in HIV infection-associated lymphoma is associated with nutritional status and is an independent risk factor for patient prognosis, which can be used as a practical predictor of patient prognosis.

## Data availability statement

The original contributions presented in this study are included in the article/supplementary material, further inquiries can be directed to the corresponding authors.

## Ethics statement

The studies involving human participants were reviewed and approved by Chongqing University Cancer Hospital. The patients/participants provided their written informed consent to participate in this study.

## Author contributions

TL and RH: conceptualization, original draft preparation, review, and editing. JiL, QL, CW, JuL, and LuX: conceptualization, supervision, review, and editing. LiX and YL: review and editing. All authors contributed to the article and approved the submitted version.
